# Comparison of Light Condition-Dependent Differences in the Accumulation and Subcellular Localization of Glutathione in *Arabidopsis* and Wheat

**DOI:** 10.3390/ijms22020607

**Published:** 2021-01-09

**Authors:** Anna Gasperl, Eszter Balogh, Ákos Boldizsár, Nadine Kemeter, Richard Pirklbauer, Stefan Möstl, Balázs Kalapos, Gabriella Szalai, Maria Müller, Günther Zellnig, Gábor Kocsy

**Affiliations:** 1 Institute of Biology, Plant Sciences, University of Graz, NAWI Graz, 8010 Graz, Austria; anna.gasperl@uni-graz.at (A.G.); nadine.kemeter@uni-graz.at (N.K.); richard.pirklbauer@uni-graz.at (R.P.); stefan.moestl@uni-graz.at (S.M.); maria.mueller@uni-graz.at (M.M.); guenther.zellnig@uni-graz.at (G.Z.); 2 Agricultural Institute, ELKH Centre for Agricultural Research, 2462 Martonvásár, Hungary; balogh.eszter@atk.hu (E.B.); akos.boldizsar@gmail.com (Á.B.); kalapos.balazs@atk.hu (B.K.); szalai.gabriella@atk.hu (G.S.)

**Keywords:** *Arabidopsis*, cell compartment, far-red light, glutathione, redox state, light intensity, wheat

## Abstract

This study aimed to clarify whether the light condition-dependent changes in the redox state and subcellular distribution of glutathione were similar in the dicotyledonous model plant *Arabidopsis* (wild-type, ascorbate- and glutathione-deficient mutants) and the monocotyledonous crop species wheat (Chinese Spring variety). With increasing light intensity, the amount of its reduced (GSH) and oxidized (GSSG) form and the GSSG/GSH ratio increased in the leaf extracts of both species including all genotypes, while far-red light increased these parameters only in wheat except for GSH in the GSH-deficient *Arabidopsis* mutant. Based on the expression changes of the glutathione metabolism-related genes, light intensity influences the size and redox state of the glutathione pool at the transcriptional level in wheat but not in *Arabidopsis*. In line with the results in leaf extracts, a similar inducing effect of both light intensity and far-red light was found on the total glutathione content at the subcellular level in wheat. In contrast to the leaf extracts, the inducing influence of light intensity on glutathione level was only found in the cell compartments of the GSH-deficient *Arabidopsis* mutant, and far-red light increased it in both mutants. The observed general and genotype-specific, light-dependent changes in the accumulation and subcellular distribution of glutathione participate in adjusting the redox-dependent metabolism to the actual environmental conditions.

## 1. Introduction

Temporal and spatial changes in light conditions occurring during the day, season and year or at various altitudes and latitudes [1] influence reactive oxygen species (ROS) production, and thus the antioxidant scavenging system, including the ascorbate-glutathione cycle [2,3,4]. Glutathione is a central component of the redox system participating in adjusting growth and development to changing environmental conditions including alterations in the intensity and spectrum of light. Excess absorbed light is damaging to plants because it can lead to the enhanced production of ROS, causing photo-oxidative stress. However, ROS can also activate various protecting mechanisms and adjust glutathione metabolism to the changes in the environment.

In the light-dependent adjustment of the size of the glutathione pool its synthesis plays a major role. This pathway depends on the availability of cysteine, glutamic acid and glycine, which are tied together by γ-glutamylcysteine synthetase (γ-ECS) and glutathione synthetase (GSHS) in two subsequent reactions. In the first step, γ-glutamylcysteine is formed by γ-ECS, then converted into glutathione by GSHS [2]. In *Arabidopsis,* localization studies have demonstrated that γ-ECS is present only in chloroplasts, while GSHS is found in both chloroplasts and cytosol. The *AtGSHS2* gene encodes two transcripts from which the most abundant one is the shorter form with cytosolic localization [5]. Both γ-ECS and GSHS have been reported to respond to light and some stress conditions (e.g., drought, certain pathogens, heavy metals) and their activation can increase the size of the glutathione pool allowing a more efficient protection against adverse environmental conditions [2].

Besides the size of the glutathione pool, the ratio of its oxidized (GSSG) and reduced (GSH) forms is also important in the fine regulation of the cellular redox environment, metabolism and stress response. Glutathione reductases (GR) mediate the conversion of GSSG into GSH and play a crucial role in the elimination of H_2_O_2_ molecules through the ascorbate–glutathione cycle. Higher plants contain cytoplasmic/peroxisomal GR and chloroplastic/mitochondrial GR. In *Arabidopsis*, cytosolic GR1 plays an essential role in H_2_O_2_ metabolism and signaling during normal conditions [6], while GR2, located in the chloroplast and mitochondria, helps to maintain the function of PSII under excess light conditions by increasing the level of GSH. This mechanism helps to avoid photo-damage by facilitating a rapid response to light stress [7,8,9]. The light spectrum-dependent modulation of GR activity was reported in cotyledons of tomato [10]. Taken together, GR participates in the maintenance of the appropriate GSSG/GSH ratio, which is crucial to protect DNA, proteins and lipids from oxidation under adverse environmental conditions [2,11].

In leaf tissues of *Arabidopsis* Col-0 wild-type plants and in the ascorbate-deficient *vtc2* mutant, total glutathione level (hereafter referred to as glutathione) significantly increased in response to high light stress and the rate of change was even higher in the case of the mutant genotypes probably to compensate for the ascorbate-deficiency [12,13,14]. Higher amounts of ascorbate and glutathione were also observed in leaves of low light-grown, tocopherol-deficient *Arabidopsis vte1* mutants in order to replace the function of tocopherol in the removal of ROS [15]. A sudden change in light conditions, affecting the electron transport between PSI and PSII, also caused a slight alteration in the total amount of glutathione in *Arabidopsis* plants [16]. The level of antioxidants (ascorbate, glutathione, tocopherol) exhibited diurnal rhythms with maximum values during midday and minimum values during the night in high alpine plant species [17], which further confirms their regulation by light. Zechmann [18] confirmed a diurnal rhythm for glutathione content in cell compartments, with the lowest values at the end of the night, maximum values after 3 h of light and a strong decrease within the next 1–2 h. Besides light intensity, the proportion of blue, red and far-red spectral components also influenced the amount of glutathione in flag leaves of wheat [19]. Both blue light and low red/far-red ratio reduced the glutathione content [4,19]. In addition, shaded leaves of bean exposed to a low red/far-red ratio had a much lower glutathione reductase (GR) activity and reduced glutathione (GSH) level compared to those ones grown in full light with equal red/far-red ratios [20,21]. Consequently, the shaded leaves exhibited a greater susceptibility to oxidative stress. These results indicate that changes in light conditions modify the size and redox state of glutathione [22], which can activate the redox-regulated protective mechanisms in plants [20,23].

Besides the alterations in glutathione content at the tissue level, its subcellular changes can also be important in the fine tuning of the adaptation to changing light conditions. Results from Heyneke et al. [3] confirm that the light intensity-dependent accumulation of ascorbate and glutathione is cell compartment-specific and differs between wild-type (Col-0) and mutant *Arabidopsis* genotypes deficient in ascorbate (*vtc2-1*) or glutathione (*pad2-1).* Impaired ascorbate and glutathione synthesis in the *vtc2-1* and *pad2-1* mutant originates from a mutation in the gene encoding γ-glutamylcysteine synthetase (γ-ECS, GSH1) and GDP-l-galactose phosphorylase, respectively. In *Arabidopsis* wild-type plants, high light exposure for a few hours increased glutathione levels in the chloroplasts and the cytosol, cell compartments associated with glutathione synthesis and the detoxification of ROS from an overstrained electron transport chain. A similar alteration was observed in peroxisomes where ROS and H_2_O_2_ originating from photorespiration are detoxified by GSH together with enzymes of the ascorbate-glutathione cycle and H_2_O_2_ degradation pathways [3]. Long-term excess light, in turn, resulted in glutathione accumulation mainly in the chloroplasts accompanied by an increase in vacuolar anthocyanins, decrease in chlorophyll and ultrastructural adaptations in the chloroplasts. While the glutathione-deficient *pad2-1* mutant compensated for the lack of glutathione at the subcellular level by accumulating more ascorbate in the peroxisomes during excess light conditions, the ascorbate deficient *vtc2-1* mutant compensated for the lack of ascorbate by accumulating more glutathione in the chloroplasts and nuclei compared to the wild-type plants, respectively. These results indicate the fine subcellular control of glutathione level, which allows the organelle-specific maintenance of the redox environment ensuring the optimal function of the redox-sensitive proteins.

We hypothesize that changes in the light intensity and spectrum may affect the antioxidant system, and thus likewise the size and redox state of the glutathione pool in the plants. The aim of the present study was the comparison of the light condition-dependent (the conditions were selected on the basis of our previous results, [4,19]) differences in the accumulation and subcellular localization of glutathione in the leaves of the dicotyledonous *Arabidopsis* and monocotyledonous wheat. Furthermore, the wild type *Arabidopsis* plants (Col-0), ascorbate- and glutathione-deficient mutants (*vtc2-1* and *pad2-1*) were also included into the experiments in order to see the effect of altered ascorbate and glutathione levels on the response to the various light conditions. Although some studies are available on the changes in glutathione levels of plant extracts after modifying the spectrum in individual species without interspecific comparisons, its effect on subcellular glutathione distribution has not been studied earlier.

## 2. Results

### 2.1. Light Condition-Dependent Differences in the Amount and Redox State of Glutathione in Leaf Extracts

**Figure 1:**
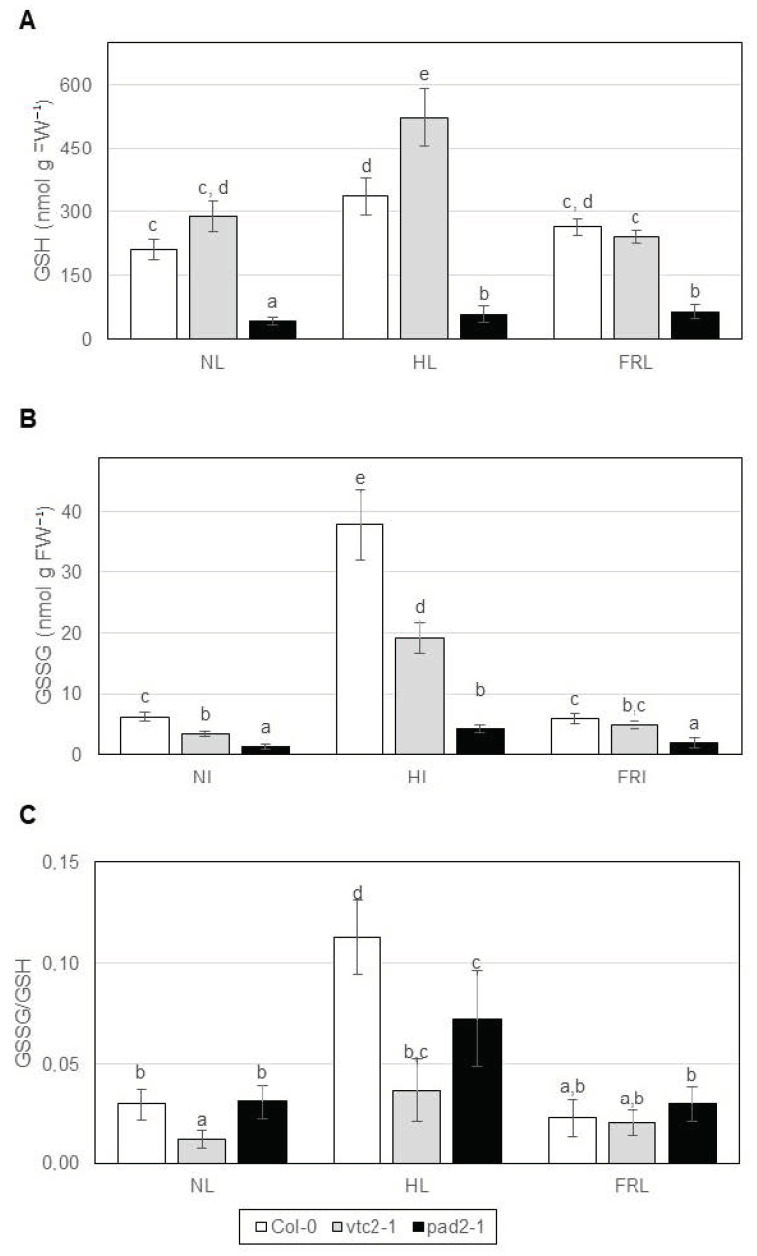
Glutathione content in leaf extracts of *Arabidopsis* lines grown under various light conditions. (**A**) Reduced glutathione (GSH), (**B**) oxidized glutathione (GSSG), (**C**) GSSG/GSH ratio. The experiment was repeated three times with three parallels. Error bars represent standard deviations (*n* = 3). Statistical analysis was performed by two-factor ANOVA. Significant differences at *p* ≤ 0.05 are indicated by different letters above the columns. NL: normal light (white light at 250 µmol m^−^² s^−1^), HL: high light (white light at 500 µmol m^−^² s^−1^), FRL: far-red light (blue/red ratio of 1:5 combined with red/far-red ratio of 10:1 at 250 µmol m^−^² s^−1^), Col-0: wild type *Arabidopsis*, *vtc2-1*: ascorbate-deficient mutant, *pad2-1*: glutathione-deficient mutant.

In all three investigated *Arabidopsis* genotypes, the amounts of GSH and GSSG and their ratio (henceforth denoted as GSSG/GSH ratio) increased (1.6- to 5-fold increase) in high light (white light at 500 µmol m^−^² s^−1^) compared to normal light (white light at 250 µmol m^−^² s^−1^); however, they were not significantly affected by far-red light (blue/red ratio of 1:5 combined with red/far-red ratio of 10:1 at 250 µmol m^−^² s^−1^) except for GSH in the glutathione-deficient *pad2-1* mutant (Figure 1). Comparing the genotypes, the GSH level was many-fold higher (4-fold at minimum) in Col-0 and the ascorbate-deficient *vtc2-1* than in *pad2-1*. Under high light, more GSH was detected in *vtc2-1* compared to Col-0. The GSSG content was the lowest in *pad2-1* compared to the other two genotypes. Both GSSG contents and GSGG/GSH ratios were increased by high light in all three genotypes. The GSSG/GSH ratio was 0.5-fold lower in *vtc2-1* than in wild type and *pad2-1* plants in normal and high lights.

**Figure 2:**
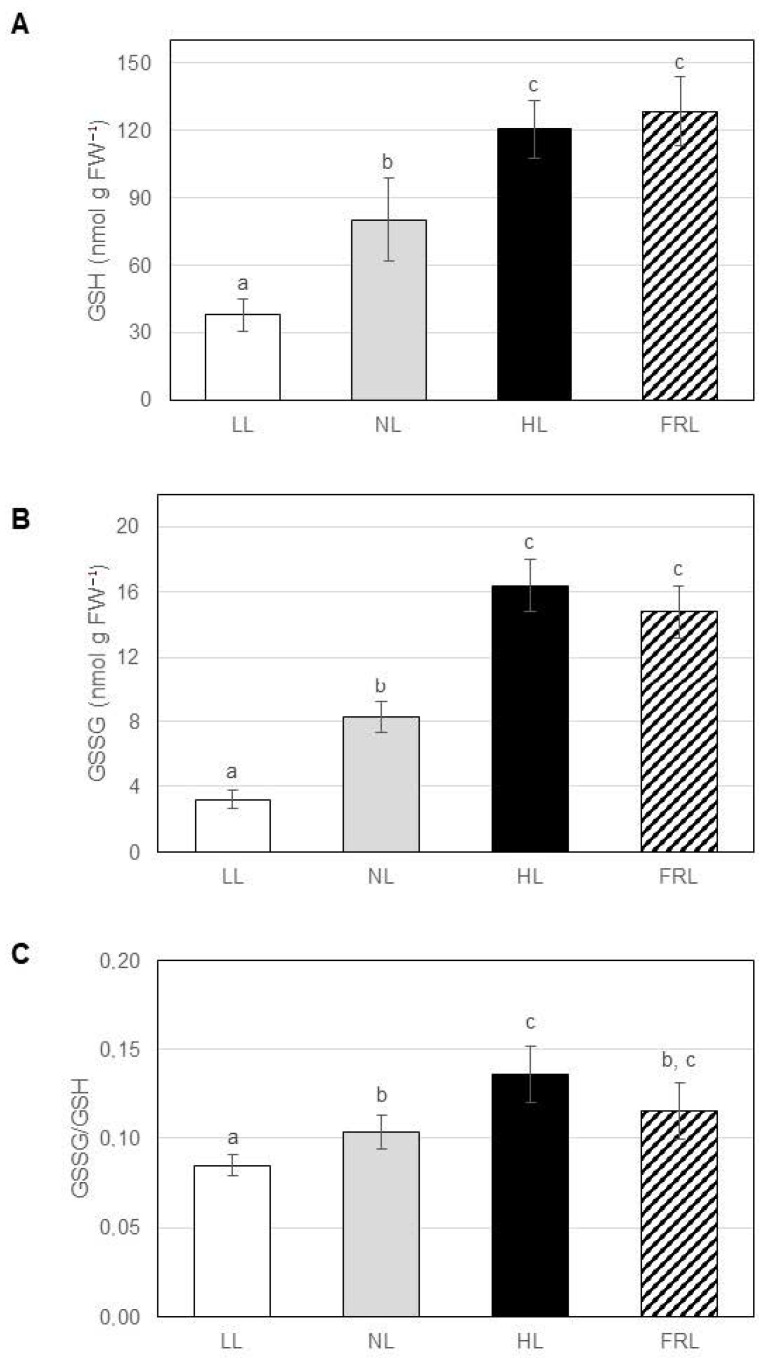
Glutathione content in leaf extracts of wheat grown under various light conditions. (**A**) reduced glutathione (GSH), (**B**) oxidized glutathione (GSSG), (**C**) GSSG/GSH ratio. The experiment was repeated three times with three parallels. Error bars represent standard deviations (*n* = 3). Statistical analysis was performed by one-factor ANOVA. Significant differences at *p* ≤ 0.05 are indicated by different letters above the columns. LL: low light (white light at 50 µmol m^−^² s^−1^), NL: normal light (white light at 250 µmol m^−^² s^−1^), HL: high light (white light at 500 µmol m^−^² s^−1^), FRL: far-red light (blue/red ratio of 1:5 combined with red/far-red ratio of 10:1 at 250 µmol m^−^² s^−1^).

In wheat, GSH and GSSG contents increased as light intensity was increased (3.3- and 5-fold difference between low (white light at 50 µmol m^−^² s^−1^) and high lights, respectively) and the same change occurred in far-red light compared to normal light (3.6- and 4.6-fold differences, respectively) (Figure 2A,B). A similar tendency was observed for the GSSG/GSH ratio only in the case of the light intensity but with smaller differences (Figure 2C). While light intensity affected the amount and redox state of glutathione in both species, far-red light influenced GSH and GSSG levels only in wheat, except for GSH in the *pad2-1 Arabidopsis* mutant.

### 2.2. Levels of Glutathione Metabolism-Related Transcripts under Various Light Conditions in Leaf Extracts

**Figure 3:**
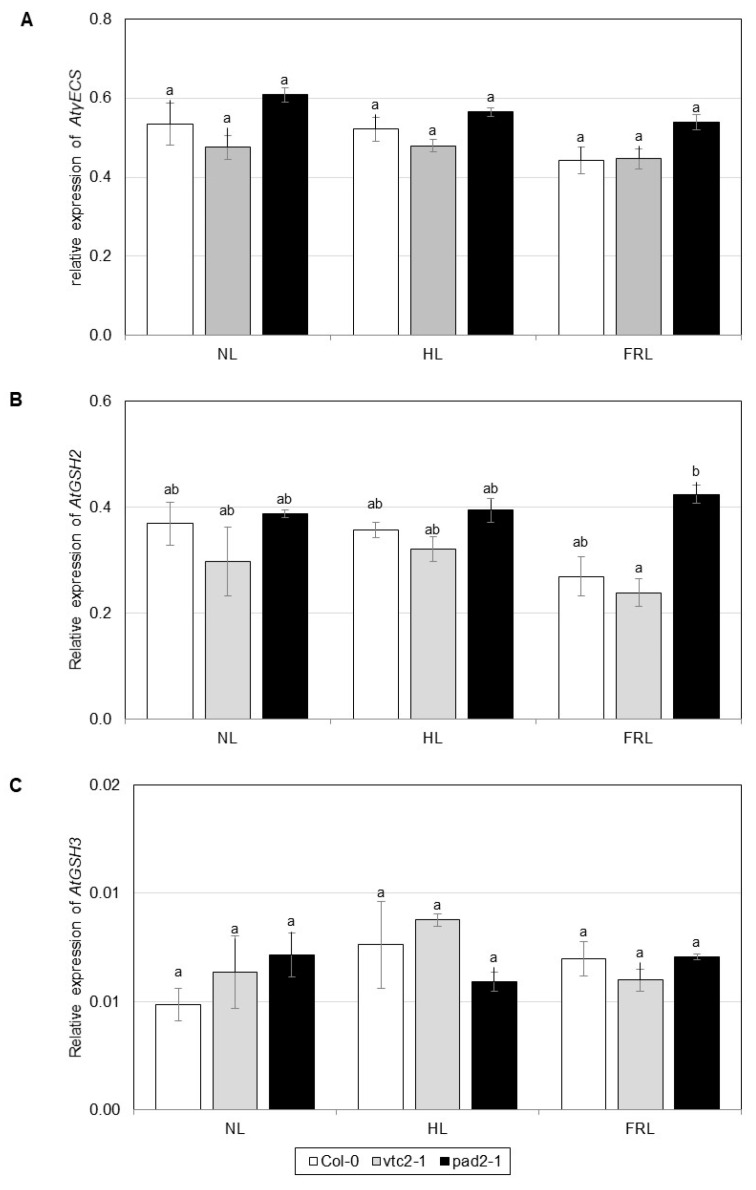
Expression of genes encoding enzymes of glutathione synthesis in leaf extracts of *Arabidopsis* lines grown under various light conditions. (**A**) γ-glutamylcysteine synthetase (*AtγECS*), (**B**) glutathione synthetase 2 (*AtGSHS2*), (**C**) glutathione synthetase 3 (*AtGSHS3*). The experiment was repeated three times with three parallels. Error bars represent standard deviations (*n* = 3). Statistical analysis was performed by two-factor ANOVA. Significant differences at *p* ≤ 0.05 are indicated by different letters above the columns. LL: low light, NL: normal light, HL: high light, FRL: far-red light.

In *Arabidopsis*, the expression of the genes encoding *At*γECS (present in the chloroplast) and *At*GSHS3 (present in the cytosol) was not affected either by the light conditions or the genotype (Figure 3A,C). The transcription of the gene encoding AtGSH2 (present in the chloroplast) was higher in *pad2-1* compared to the other two genotypes in far-red light (Figure 3B). The expression of AtGSH3 was one order of magnitude smaller compared to AtGSH2. The transcript levels of the *AtGR1* (the encoded enzyme is present in the cytosol and peroxisomes) and *AtGR2* (the encoded enzyme is present in the chloroplast and mitochondria) genes were not significantly different in the three genotypes under any light conditions (Figure S1).

**Figure 4:**
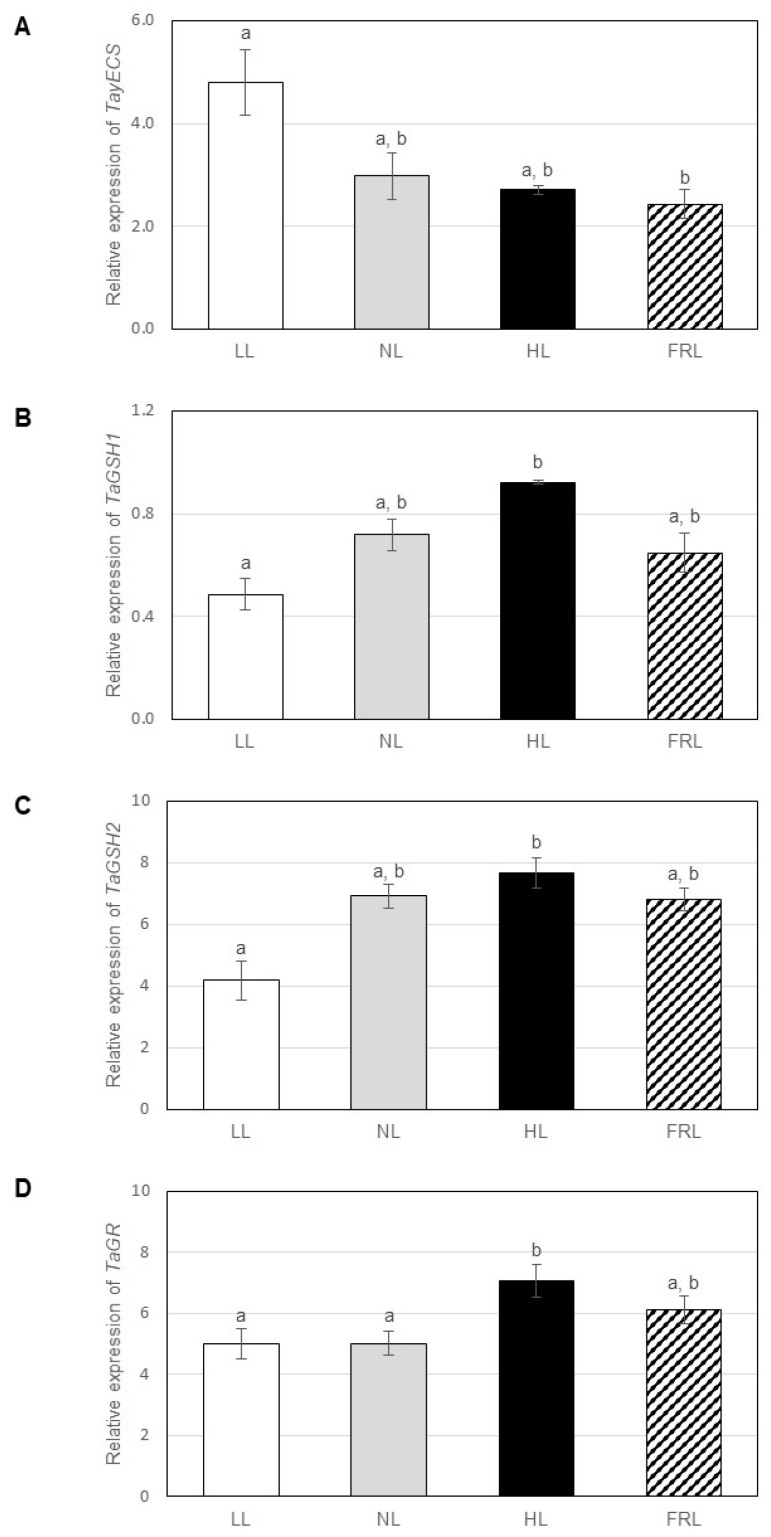
Expression of genes encoding enzymes of glutathione metabolism in leaf extracts of wheat grown under various light conditions. (**A**) γ-glutamylcysteine synthetase (*Ta*γ*ECS*), (**B**) glutathione synthetase 1 (*TaGSHS1*), (**C**) glutathione synthetase 2 (*TaGSHS2*), D: glutathione reductase (*TaGR*). The experiment was repeated three times with three parallels. Error bars represent standard deviations (*n* = 3). Statistical analysis was performed by one-factor ANOVA. Significant differences at *p* ≤ 0.05 are indicated by different letters above the columns. LL: low light, NL: normal light, HL: high light, FRL: far-red light.

In wheat, the expression of the gene encoding *Ta*γECS (present in the chloroplast) exhibited significantly lower expression in far-red light compared to low light (Figure 4A). The transcript levels of *Ta*GSHS1 (the encoded enzyme is present in the chloroplast), *Ta*GSHS2 (the encoded enzyme is present in the cytosol) and *T*aGR (the encoded enzyme is present in the cytosol) genes grew with increasing light intensity (1.4- to 1.8-fold differences between low and high lights) but were not affected by far-red light compared to normal white light (Figure 4B–D). The expression of *Ta*GSHS1 was one order of magnitude lower compared to *Ta*GSHS2. In contrast to *Arabidopsis*, light conditions affected the expression of all studied GSH metabolism-related genes in wheat.

### 2.3. Control of the Subcellular Glutathione Distribution by Light in Leaves

**Figure 5:**
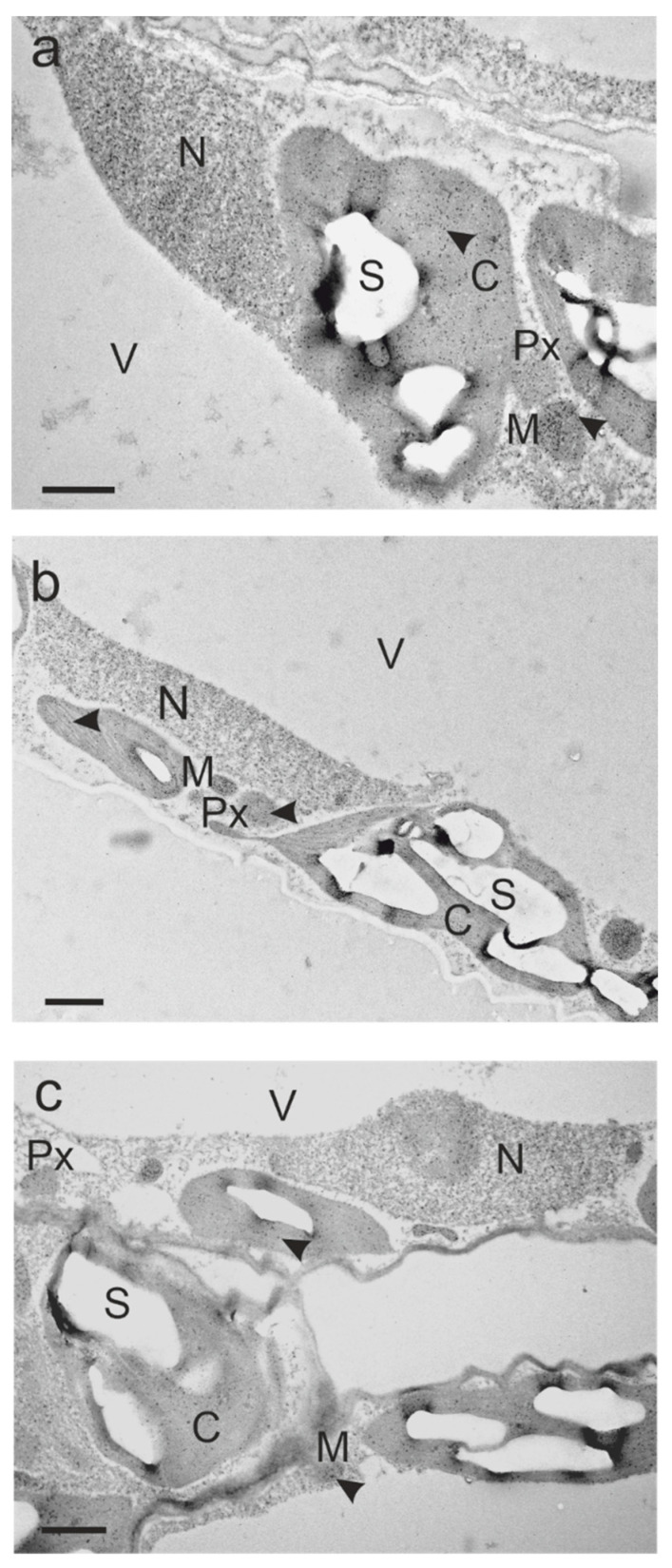
Subcellular distribution of glutathione in *Arabidopsis thaliana* Col-0 grown under various light conditions. Representative TEM images show compartment-specific glutathione distribution (dark dots, arrowhead) in parts of mesophyll cells from plants exposed to (**a**) normal light (NL), (**b**) high light (HL) and (**c**) far-red light (FRL). C = chloroplasts with starch (S), M = mitochondria, N = nuclei, Px = peroxisomes, V = vacuoles. Bars =1 µm.

**Figure 6:**
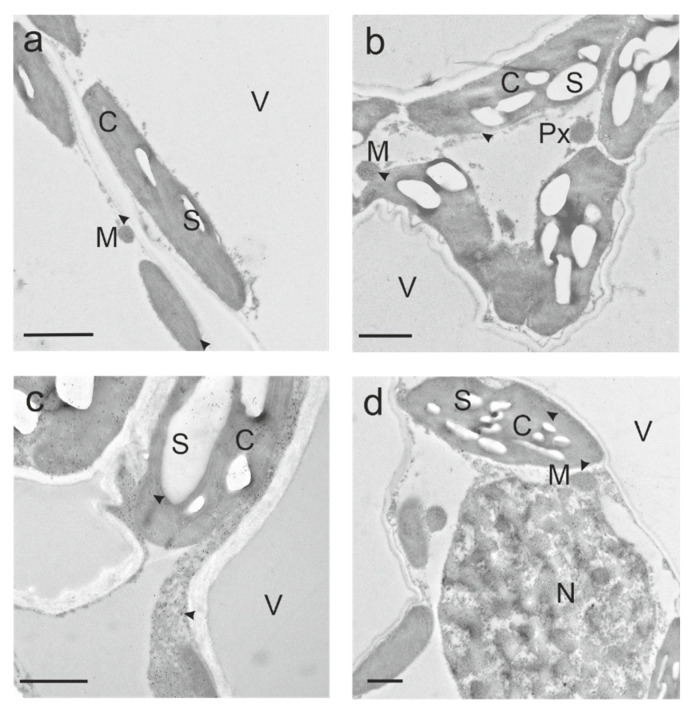
Subcellular distribution of glutathione in wheat grown under various light conditions. Representative TEM images show compartment-specific glutathione distribution (dark dots, arrowhead) in parts of mesophyll cells from plants exposed to (**a**) low light (LL), (**b**) normal light (NL), (**c**) high light (HL) and (**d**): far-red light (FRL). C = chloroplasts with starch (S), M = mitochondria, N = nuclei, Px = peroxisomes, V = vacuoles. Bars =1 µm.

Subcellular glutathione labeling density was detected by transmission electron microscopy (TEM). The distribution of glutathione-specific gold labeling in *Arabidopsis* wild-type plants (Figure 5 and Figure S5, Table 1), *vtc2-1* (Table 1, Figures S3 and S5) and *pad2-1* (Table 1, Figures S4 and S5) mutants grown under various light conditions, was similar to previous studies in dicotyledonous plants [3,24]; however, overall glutathione labeling density was lower than previously published for the *vtc2-1* mutant. In wheat plants, the distribution and labeling density of glutathione-specific gold labeling was likewise comparable to previously published results in dicotyledonous plants (Figure 6 and Figure S6, Table 1).

At the subcellular level, *Arabidopsis* genotypes responded differently to high light and far-red light (Figure 5a–c, Figures S3a–c, S4a–c and S5A–E, Table 1). As with the results found for GSH and GSSG levels in leaf extracts of all genotypes, glutathione labeling density significantly increased: 5-fold in nuclei, 2-fold in peroxisomes and the cytosol and less remarkably in mitochondria (1-fold) and chloroplasts (+59%) in *Arabidopsis pad2-1* plants grown under high light compared to white normal light. In contrast to GSH and GSSG levels in leaf extracts, far-red light affected the glutathione labeling density in the same cell compartments similarly to high light in *pad2-1* plants: significant 2-fold increase in nuclei, 1-fold increase in peroxisomes and the cytosol, significant +51% and +35% increase in mitochondria and chloroplasts, respectively. With the exception of a significant increase in the mitochondria (+27%), subcellular glutathione level was unaffected (in peroxisomes) or significantly decreased (by −27% in nuclei, −24% in chloroplasts and −16% the cytosol) by high light in Col-0 plants, while far-red light led to a slight, significant decrease in glutathione labeling density in nuclei (−18%) and chloroplasts (−12%) compared to normal light. A minor response to high light was found in the *vtc2-1* mutant, in which solely the cytosol glutathione labeling density decreased significantly by 21% compared to normal light. *Arabidopsis vtc2-1* plants responded similarly to far-red light as *pad2-1* plants, showing a significant increase in the same compartments, except for mitochondria: 1.8-fold in peroxisomes, +78% in chloroplasts, +37% in nuclei and +32% in the cytosol. Considering the genotypes, subcellular glutathione labeling density was—irrespective of the light treatment—the highest in Col-0, followed by *vtc2-1* and *pad2-1* (Figure 5 and Figures S3–S5, Table 1). Corresponding to GSH and GSSG extract levels in wheat, the subcellular glutathione labeling density significantly grew in wheat with increasing light intensity and in plants grown in far-red light compared to normal light (Figure 6a–d and Figure S6A–E, Table 1). Only in the chloroplasts of wheat grown under low light conditions the glutathione concentration was not significantly different from plants grown under normal light. The strongest effect in glutathione labeling density, with respect to increasing light intensity, was found in the peroxisomes (48% significant decrease in LL compared to NL, 1.4-fold significant increase in HL compared to NL), the cytosol (65% significant decrease in LL compared to NL, 1-fold significant increase in HL compared to NL) and nuclei (78% significant decrease in LL compared to NL, 86% significant increase in HL compared to NL), a modest, significant increase in mitochondria (24% significant decrease in LL compared to NL, 27% significant increase in HL compared to NL). The significant increase in glutathione in wheat plants grown under far-red light was the highest compared to normal light in the cytosol (1.8-fold), followed by chloroplasts (1.5-fold), nuclei and peroxisomes (1-fold), and again, a modest increase in mitochondria (+38%). The glutathione labeling density of plants grown in normal light was higher in *Arabidopsis* Col-0 compared to wheat: mitochondria ~1-fold, peroxisomes ~2-fold, chloroplasts ~6-fold, cytosol ~2-fold, nuclei ~2-fold (Figure 5, Figure 6, Figures S5 and S6, Table 1).

## 3. Discussion 

### 3.1. Light Condition-Dependent Differences in the Glutathione Accumulation in Leaf Extracts

The observed similar increase in GSH content in leaf extracts of *Arabidopsis* (varied between 1.4 and 1.8-fold in the three genotypes) and wheat (1.5-fold) in high light compared to normal light indicates the activation of the antioxidant system, which will facilitate the efficient removal of ROS produced in higher concentrations in high light [25]. However, a more oxidizing redox environment of the leaf tissues was found in high light in both plant species, because of the simultaneous larger increase in the GSSG content, resulting in a higher ratio of GSSG in relation to GSH (henceforth denoted as GSSG/GSH ratio). Interestingly, the change in this parameter was much more pronounced in *Arabidopsis* than in wheat (1.5-fold for GSSG and 2.5- to 4.5-fold for the GSSG/GSH ratio). Despite this difference in the change in GSSG content and GSSG/GSH ratio, still enough GSH was available for the various metabolic processes under high light conditions in *Arabidopsis*, due to its 2-fold higher GSH content compared to wheat, except for the GSH-deficient *pad2-1* mutant. This difference between the two species may result from a higher GSH synthesis or smaller GSH degradation rate in *Arabidopsis.*

As was the case with *Arabidopsis* and wheat, the GSH content also depended on the available light intensity in bean leaves, as it was indicated by its decrease after a short period of plant cultivation at lower light intensity [21]. This change was accompanied by a reduction of the glutathione reductase activity, which can still be sufficient for the proper function of the ascorbate-glutathione cycle because of the reduced formation of H_2_O_2_. It was also shown in *Arabidopsis*, that the glutathione levels were adjusted very rapidly to altered light conditions: only a 90 s exposure to high light resulted in increased GSH and GSSG contents [9]. The increase was derived from the activation of its synthesis as shown by the parallel increase in the level of its precursors, Cys and Gly. The light intensity-dependent accumulation of the third GSH precursor, Glu, was observed in wheat seedlings [4]. The induction of GSH synthesis by high light was also shown in the flag leaves of wheat based on the accumulation of the intermediate product, γ-glutamylcysteine [19]. Light intensity may influence the size and redox state of glutathione at the transcriptional level as shown for wheat in the present study. However, in other species, similarly to *Arabidopsis* in this study, the changes in GSH and GSSG contents may not be accompanied by expression changes of the genes encoding the related enzymes of their metabolism. Thus, light intensity controls the size and redox state of the glutathione pool by different mechanisms including posttranscriptional regulatory mechanisms in the various species.

In contrast to light intensity, far-red light induced an increase in the amount of GSH and GSSG only in the leaf extracts of wheat, except for GSH in the *pad2-1 Arabidopsis* mutant. This difference can be explained by the much smaller content of GSH in wheat (0.3 to 0.4-fold smaller) and the *pad2-1 Arabidopsis* mutant (0.1 to 0.2-fold smaller) compared to the other two *Arabidopsis* genotypes. Thus, the sensitivity of the GSH metabolism (synthesis and degradation) to far-red light may depend on the amount of GSH. A higher ratio of FR did not influence the GSH content at the transcriptional level in wheat and the *pad2-1 Arabidopsis* mutant since the expression of the related genes did not increase in FR light, and in the case of γECS in wheat even decreased, which indicates a posttranscriptional regulation. While the increase in R/FR ratio increased the GSH content in wheat and the *pad2-1 Arabidopsis* mutant, its decrease reduced the GSH content in bean [20]. In addition, the supplemental FR light also induced a greater GSH accumulation in barley ( Boldizsár, ELKH Centre for Agricultural Research, Martonvásár, Hungary, modification of glutathione content by far-red light in barley, 2018), which further corroborates the control of GSH levels by far-red light. The effect of spectrum (blue and red lights) on GSH metabolism was also shown in tomato [10]. The spectrum-dependent alterations in the GSH content may also derive from the modification of the activity of glutathione *S*-transferase, catalyzing the conjugation of GSH by xenobiotics and various other compounds (reviewed in [26]). The expression of the gene encoding this enzyme was affected by blue and far-red light in wheat [4]. In addition, the interaction of a glutathione *S*-transferase with far-red insensitive 219 protein, which is involved in phytochrome A-mediated signaling, was demonstrated in *Arabidopsis* [27], giving further evidence for the control of GSH metabolism by far-red light.

The changes in the redox state of glutathione observed in leaf tissue of wheat in high and far-red light may modify the expression and activity of redox responsive genes and proteins, respectively [28] and also the metabolite profile [29]. These light-dependent processes also contribute to the successful adaptation to stress conditions [30]. Hence, light intensity-dependent changes in GSH content affected the adaptation of wheat to drought [31]. The present results about the effect of spectrum on glutathione can be useful for the selection of more adaptable crop genotypes, which can be cultivated at high altitudes and at latitudes having special spectral conditions [1].

### 3.2. Control of the Subcellular Total Glutathione Distribution by Light in Leaves

Although the light intensity- and spectrum-dependent changes in GSH and GSSG levels and their ratio in leaf extracts indicate the importance of GSH and GSSG in the adaptation to the various environmental conditions, a deeper insight into their fine regulatory functions can be obtained through the study of the subcellular distribution of glutathione. The differences both in the size and redox state of the glutathione pool in individual cell compartments have an important role in the control of the local subcellular redox environment and gradient affecting many redox-dependent metabolic processes [32].

The effect of high light on the subcellular distribution of glutathione (GSH + GSSG) depended on the duration of the exposure in *Arabidopsis* wild-type (Col-0) plants as reported by Heyneke et al. [3]. An increase in glutathione in chloroplasts, peroxisomes and the cytosol was reported as a reaction to short term (4 h) high light (300 and 700 µmol m^−^² s^−1^ respectively, compared to control light conditions’ 150 µmol m^−^² s^−1^). However, when the plants were exposed to a longer period (14 days) of high light, Col-0 plants seemed to adapt and showed elevated glutathione levels most prominently in chloroplasts, and a modest increase of glutathione in the mitochondria only at 300 µmol m^−^² s^−1^ light intensity. Interestingly, we found a comparable modest accumulation of mitochondrial glutathione levels and even a slight decrease in chloroplast, nuclei and cytosol glutathione levels in high light (500 µmol m^−^² s^−1^) after four days in *Arabidopsis*. Heyneke et al. [3] proposed an acclimation process during high light exposure based upon their results for short- and long-term high light effects on the subcellular glutathione (and ascorbate) pool. Considering that, our results for subcellular glutathione distribution in Col-0 plants after four days under a high light regime may represent an intermediate stage in this acclimation process. In the *pad2-1* mutant, reduced glutathione (GSH) levels in leaf extracts are many-fold lower compared to Col-0 (present study, [33]). However, at the subcellular level, *pad-2-1* was found to be capable of accumulating glutathione in mitochondria in the same range as Col-0, yet in all other cell compartments its level was up to one order of magnitude lower [3,34,35,36,37,38]. In the present study the glutathione level was between 3-fold and 5-fold lower in mitochondria and between 20-fold and 200-fold lower in all other cell compartments of *pad2-1* compared to Col-0. While Heyneke et al. [3] reported lower glutathione levels in mitochondria, nuclei, peroxisomes and the cytosol of *pad2-1* plants after long term high light exposure, we found a distinct increase in glutathione levels in the same compartments and a moderate increase in the chloroplasts of *pad2-1* plants exposed for four days to a light intensity of 500 µmol m^−^² s^−1^. These contradicting reactions may again be explained by an intermediate stage of adaptation to increased light intensity. The glutathione-specific labeling density in the ascorbate deficient *vtc2-1* mutant was previously reported to be close to the labeling density in Col-0 plants in all cell compartments [3], which corresponds to our results for the GSH content in the leaf extracts. Unexpectedly, the subcellular glutathione level in the *vtc2-1* mutant was found to be in the same range as in the *pad2-1* mutant under the present experimental conditions. This difference can be explained by the different growth conditions and developmental stage of the plants in the two experiments. Considering that the GSSG/GSH ratio in the leaf extracts was lowest in *vtc2-1* in the present study under high light after four days, these plants did not suffer from oxidative stress. Accordingly, high light had no effect on the subcellular glutathione levels except for a modest decrease in the cytosol.

The possible effects of far-red light exposure on subcellular glutathione distribution have not been reported earlier in plants. As observed for GSH and GSSG levels in leaf extracts of the three investigated *Arabidopsis* genotypes (except for GSH in *pad2-1*), subcellular glutathione labeling density was largely unaffected by far-red light in wild-type plants. However, it increased in all compartments of *pad2-1* and *vtc2-1* mutants except for the mitochondria of *vtc2-1* plants. Considering the aforementioned hypothesis for plants going through an acclimation process in response to a change in light intensity, certain changes in the glutathione contents of the glutathione- and ascorbate- deficient mutants (*pad2-1* and *vtc2-1*) during exposure to high light or far-red light may occur asynchronously at the tissue and the subcellular level or may be limited under our experimental conditions due to lower availability of glutathione and ascorbate. Given that balancing redox homeostasis is a highly dynamic process in plants ([39] and references therein), unchanged glutathione concentrations at the tissue level during far-red light exposure may result from special high turnover and transport outside of the five studied cell compartments [40,41].

To the best of our knowledge, this study is the first to provide subcellular glutathione-specific labeling in a monocotyledonous plant. The highest amount of glutathione was found in the mitochondria of wheat after four days of exposure to different light intensities, which corresponds to earlier studies in dicotyledonous plants [24,37]. Mitochondrial glutathione content showed a moderate response both to low and high light. A similar trend involving the same light treatments has been reported earlier in wheat leaf extracts for the glutathione precursor cysteine [4] and it corresponds well with our present and previously published results for *Arabidopsis* Col-0 [3]. In peroxisomes, cytosol and nuclei of wheat a marked decrease in glutathione was found under low light, whereas glutathione increased considerably under high light exposure. In contrast, chloroplast glutathione levels were ranging roughly at the same level under low and normal light intensity, while high light resulted in a 1-fold increase, which is in agreement with the findings of Heyneke et al. [3] for *Arabidopsis* Col-0 (see above). Although in wheat the same cell compartments exhibited an increase in glutathione when exposed to high light (except for chloroplasts) and far-red light, the greatest increase under far-red light was found in the cytosol and not in the peroxisomes, followed by the chloroplasts and nuclei in *Arabidopsis*. Heyneke et al. [3] proposed that an increase of glutathione in chloroplasts of *Arabidopsis* wild-type (Col-0) plants with increasing light intensity may reflect successful adaptation to ROS formation. This hypothesis may be likewise applied to the results of high light- and far-red light-treated chloroplasts of wheat in the present study. Given that the authors [3] found a strong decrease in chloroplast glutathione content of *Arabidopsis* during exposure to high light at 1500 µmol m^−^² s^−1^, the increase in chloroplast glutathione level at 500 µmol m^−^² s^−1^ exposure appears to be in the physiological range of *Arabidopsis* Col-0 and wheat.

Immunogold labeling is a high-resolution approach, which allows sub-organellar localization of glutathione, for example, in the thylakoid lumen under high light stress (Figure 5b) [3]. However, up to the present date, there is a lack of specific antibodies for the reduced and oxidized forms of glutathione. Meanwhile, stable transformation of *Arabidopsis* with roGFP (reduction-oxidation sensitive GFP) fused to organelle- or membrane-specific targets has proven to be a useful alternative approach in discriminating reduced and oxidized forms of glutathione at the subcellular level ([42] and references therein, [43]). While extended darkness or elicitors affected the redox state of peroxisomes, high light (three hours) or drought affected the redox state of mitochondria and chloroplasts, which is in line with immunolabeling results for chloroplasts in short term (4 h) high light-treated *Arabidopsis* wild-type plants [3]. In addition, Haber et al. [42] report a rapid oxidation of chloroplastic roGFP, which was more severe at high light intensities of 750–1700 µmol m^−^² s^−1^ compared to 220–650 µmol m^−^² s^−1^, upon the onset of high light treatment followed by gradual reduction. The future production of roGFP-containing monocots including wheat would be important in order to make general statements about the subcellular regulation of the redox state of glutathione by light and other environmental factors.

## 4. Materials and Methods

### 4.1. Plant Material and Growth Conditions

Seeds of *Arabidopsis thaliana* [L.] Heynh. ecotype Columbia (Col-0), the glutathione- and ascorbate-deficient mutants *pad2-1* and *vtc2-1*, respectively, were sown in sterile garden soil and cultivated in a growth chamber (Conviron PGR15; Controlled Ev., Ltd., Winnipeg, Canada) for 9 days at 23/22 °C (day/night temperature) and 70/75% (day/night) relative humidity under short day conditions with 8 h of light (starting at 8:00 am) at 120 µmol m^−^² s^−1^. After this initial growth, 30 pots (referring to 10 pots per individual light treatment) containing two plants each per ecotype/mutant were cultivated in the growth chamber for 13 days at 21/18 °C (day/night temperature) and 75% relative humidity (day and night) under short day conditions with 8 h of light at 250 µmol m^−^² s^−1^. For the following 13 days of cultivation, only the light regime has been changed to long day conditions with 16 h of light (starting at 00:00 am); all other conditions remained the same.

Seeds of *Triticum aestivum* L. ssp. *aestivum* variety Chinese Spring were grown according to Toldi et al. [4]. Briefly, seeds were germinated for one day at 25 °C, for three days at 4 °C and for two days again at 25 °C in petri dishes between wet filter papers in the dark. Seedlings were grown at 20/17 °C (day/night temperature) and 75% relative humidity with 16 h of light (starting at 00:00 am) at 250 µmol m^−^² s^−1^, in 12 plastic pots. Each pot contained 15 seedlings (referring to three pots per individual light treatment). They were grown on half-strength Hoagland solution [44], which was changed every second day, for 10 days.

### 4.2. Different Light Treatments

After 35 days of growth, *Arabidopsis* plants were subjected to three different light conditions for four days as described previously [4]: white light at 250 µmol m^−^² s^−1^ (referred to as normal light); white light at 500 µmol m^−^² s^−1^ (referred to as high light) and far-red light (blue/red ratio of 1:5 combined with red/far-red ratio of 10:1) at 250 µmol m^−^² s^−1^ (Table S1). Light modules were equipped with a continuous wide spectrum LED (Philips Lumileds, LXZ2-5790-y, Amsterdam, The Netherlands) and three narrow spectrum LED armatures with dominant wavelengths of 448 nm (Philips Lumileds, LXZ1-PR01, Amsterdam, The Netherlands), 655 nm (Philips Lumileds, LXZ1-PA01, Amsterdam, The Netherlands) and 750 nm (Edison Edixeon, 2ER101FX00000001, Moers, Germany). After 15 days of growth (including germination), wheat seedlings were grown under the three light conditions described for *Arabidopsis* for four days. Additionally, the effect of a 4th light condition, 50 µmol/m^2^/s intensity white light (referred to as low light), was also studied.

### 4.3. Sampling

In order to eliminate the effects of daily rhythms, young *Arabidopsis* leaves of the same size or the second fully developed leaf from the shoot tip of the wheat seedlings were sampled during the middle of the photoperiod. One part of the harvested leaves was immediately subjected to embedding for *in situ* glutathione content detection (*n* = 3). The remaining plant material was aliquoted, snap-frozen in liquid nitrogen and stored at −80 °C until further use for RNA and thiol extraction and analysis.

### 4.4. Measurement of Reduced (GSH) and Oxidized (GSSG) Glutathione

Reduced (GSH) and oxidized (GSSG) forms of glutathione were measured in a leaf sample of 200 mg as described by Kocsy et al. [45], according to the method of Kranner and Grill [46]. They were separated by reverse-phase HPLC (Waters, Milford, MA, USA) and detected by a W2475 scanning fluorescence detector (Waters, Milford, MA, USA).

### 4.5. Analysis of the Expression of the Genes Encoding Enzymes with a Glutathione Metabolism

Total RNA was extracted from the leaves using the Direct-zol™ RNA Miniprep Kit (Zymo Research, Irvine, CA, USA) according to the instructions of the manufacturer. Reverse transcription was performed using M-MLV Reverse Transcriptase and oligo(dT)15 primer (Promega, Dübendorf, Switzerland) as described by the manufacturer. The gene expression levels were determined by real-time qRT-PCR using a CFX96 thermocycler (Bio-Rad, Dreieich, Germany) with the primers summarized in Table S2 (own design, [4,31,33]). For comparison, fold change (2ΔΔCq, where ΔCq = Cq(ref) − Cq(target)) was determined [47] using *actin2* and *Ta30797 (*similar to phosphogluconate dehydrogenase protein) gene reference in *Arabidopsis* and wheat, respectively [48,49].

### 4.6. Sample Preparation for In Situ Glutathione Content Analysis via Immunohistochemical Labeling

Samples for determination of *in situ* compartment-specific glutathione content *via* immunohistochemical labeling were prepared according to Zechmann, Mauch et al. [37] and Zechmann and Müller [24]. For *Arabidopsis* and wheat samples, leaf base and leaf apex were removed. Sections of 1.5 mm² of the remaining central part of each leaf from at least three different plants were made on a modeling wax plate in 2.5% paraformaldehyde and 0.5% glutaraldehyde in 0.06 M Sørensen buffer and immediately fixed in 2.5% paraformaldehyde and 0.5% glutaraldehyde. Next, samples were rinsed in buffer, dehydrated in increasing concentrations of acetone and gradually infiltrated with increasing concentrations of LR-White resin. As a last step, samples were polymerized in fresh, pure LR-White resin at 50 °C for 48 h under anaerobic conditions. Polymerized samples were sectioned with a Reichert Ultracut S ultramicrotome (Leica Microsystems, Vienna, Austria) to receive 80 nm ultrathin sections (see Figure S2 for subcellular compartmentation in *Arabidopsis* Col-0 (a) and wheat (b) mesophyll cells of plants exposed to normal light conditions: white light at 250 µmol m^−^² s^−1^ without glutathione specific immunohistochemical labeling).

Glutathione immunohistochemical labeling was performed on coated nickel grids according to Zechmann et al. [37]. The primary, glutathione-specific antibody allows to detect total glutathione (sum of free reduced and oxidized glutathione, according to Millipore Merck KGaA) and has been proven not to target a number of displacers [37]. Care was taken that the final dilution of primary and secondary antibodies used in this study showed a minimum of background labeling outside the sample with a maximum of specific labeling in the sample. An ideal dilution of primary (glutathione x rabbit polyclonal antibody IgG, Millipore Merck KGaA, Darmstadt, Germany) and secondary (EM goat anti rabbit IgG 10 nm immunogold conjugate, British BioCell International, Cardiff, UK) antibodies has been determined in previous studies by evaluating the labeling density after a series of labeling experiments.

To ensure the specificity of the immunogold-labeling procedure, negative controls were incubated with: (i) the gold conjugated secondary antibody (goat anti rabbit IgG) without prior incubation of the section with the primary antibody, (ii) a non-specific secondary antibody (goat anti rat IgG), (iii) pre-immune serum instead of the primary antibody and (iv) the primary antibody against glutathione pre-adsorbed with an excess of reduced or oxidized glutathione, respectively, for 2 h prior to the labeling of the sections. For the latter, a solution containing either 10 mM of reduced or oxidized glutathione was incubated with 0.5% glutaraldehyde for 1 h, then its excess was saturated by incubation for 30 min in a solution of 1% (*w*/*v*) BSA. The resulting solution was used in independent experiments to saturate the glutathione antibody for 2 h prior to its use in the immunogold-labeling procedure described above. Labeling on sections treated as negative controls showed no or only very little gold particles bound to glutathione, which was similar to previous results obtained by using the same methods in different plant species [24].

Samples were blocked with 1% bovine serum albumin (BSA, Sigma-Aldrich, St. Louis, MO, USA) in phosphate-buffered saline (PBS) at pH 7.2 for 20 min at room temperature. After removal of excess blocking solution, samples were treated with the primary antibody against glutathione diluted 1:50 in PBS containing 1% goat serum for 2 h at room temperature. After a short rinse in PBS (3 times for 5 min) the samples were incubated with a 10 nm gold-conjugated secondary antibody diluted 1:50 in PBS for 90 min at room temperature. Again, samples were rinsed briefly (3 times for 5 min) in PBS and distilled water (2 times for 5 min) and post-stained with uranyl-acetate for 15 sec at room temperature to enhance contrast within the specimen to facilitate the distinction of cell compartments during TEM imaging.

### 4.7. TEM Imaging and Quantitative Analysis of Compartment-Specific Glutathione Levels

TEM imaging and quantification of compartment-specific glutathione levels were carried out as described previously [3]. Grids were examined in a Zeiss Libra 120 Plus TEM (Carl Zeiss AG, Oberkochen, Germany), and micrographs of immunogold-labeled sections of mesophyll cells were taken randomly with a Tröndle TRS 2k bottom mount camera (Tröndle, Moorenweis, Germany) or with an XF416 4k camera (Tietz Video and Image Processing Systems GmbH, Gauting, Germany).

Cell compartments (mitochondria, peroxisomes, chloroplasts, cytosol, nuclei) were visually identified, manually traced and gold particles counted automatically using the software package CellSens Dimension (OLYMPUS Life Science Europa SE & CO. KG, Hamburg, Germany). Unspecific background labeling was determined on the sections (outside the specimen) and subtracted from the values obtained in the sample. At least 11 for peroxisomes, or 60 (other cell compartments/organelles) sectioned cell compartments were analyzed for gold particle density per light treatment and ecotype/mutant for *Arabidopsis* or per light treatment for wheat samples.

### 4.8. Statistical Analysis

The statistical evaluation of thiol contents and gene expression results was carried out as described earlier [4]. Data from three independent experiments with standard deviations are shown in the figures. After checking of the prerequisites, the data sets were compared by single-factor ANOVA using IBM SPSS Statistics 22.0 (2013). Normality of the data was controlled by the Kolmogorov–Smirnov test (when *p >* 0.05, normal distribution) and homogeneity of variance was verified by the Levene test. If the variances were equal (*p >* 0.05), Tukey’s b test was used; if they were not equal (*p <* 0.05), Dunnett’s T3 post hoc test was applied.

By the *in situ* compartment-specific studies the mean amount of compartment-specific glutathione (with standard error), represented by the number of gold particles per µm² of traced cell compartment, was statistically evaluated in IBM SPSS Statistics 26.0 (2019)). First, a Shapiro–Wilk test [50] of normality was performed. Since a normal distribution of the samples was rejected (*p <* 0.05), in *Arabidopsis,* significant differences between control treatment (normal light) and the other treatments (high light, far-red light) were assessed within the same line (Col-0, *vtc2-1, pad2-1*) by the Man–Whitney U-test (asterisks above the means indicate significant difference at *p* < 0.01 **; *p* < 0.001 ***) [51]. In wheat, significant differences between treatments (low light, normal light, high light) were assessed by one-sided ANOVA according to Kruskal–Wallis [52], followed by a post-hoc comparison using the Bonferroni–Dunn approach [53] (different lower case letters above the means indicate significant differences at the 0.05 level of confidence).

## 5. Conclusions

The light intensity-induced accumulation of GSH and GSSG in the leaf tissues of various plant species is probably a general phenomenon based on the obtained similar results in *Arabidopsis* and wheat (Table S3). However, at the subcellular level, genotype-specific regulatory mechanisms may exist since the total glutathione content increased in all five examined subcellular compartments of wheat, while a similar result among the three studied *Arabidopsis* lines was only obtained in the *pad2-1* mutant. The spectrum had different effects in the leaf extracts of the two species since far-red light increased the GSH and GSSG contents only in wheat except for GSH in *pad2-1* plants. At the subcellular level, a similar difference exists taking into account only the wild-type *Arabidopsis* plants. However, in *vtc2-1* and *pad2-1* mutants, far-red light induced the accumulation of glutathione in the cell compartments. Consequently, besides the general light-dependent mechanisms of the control of glutathione accumulation and subcellular distribution, genotype-specific control processes also occur. For the determination of general differences in the effect of light on the size and redox state of glutathione pool between monocot and dicot plants, more species should be investigated from both groups. The observed regulation of the amount and redox state of glutathione by light can be the basis for the activation of the GSH-dependent protective mechanisms of crops cultivated under controlled conditions by the appropriate adjustment of the illumination or in the field by choosing crop genotypes capable of adapting to special light conditions in order to reduce stress-induced damages and consequently to increase productivity.

## Figures and Tables

**Table 1:** Compartment-specific means of gold particles bound to glutathione (± SE and percent/fold change to NL) per µm² in *Arabidopsis* wild type plant (Col-0), ascorbate deficient mutant (*vtc2-1*), glutathione deficient mutant (*pad2-1*) and wheat (Wheat). Values represent the mean of 60 cell compartments, except >11 for peroxisomes. Significant differences between NL and the other treatments (HL, FRL) were compared in *Arabidopsis* within the same line (Col-0, *vtc2-1*, *pad2-1*) by the Mann–Whitney U-Test. Significance is indicated at the 0.01 (**) or 0.001 (***) level of confidence. Wheat: Different lowercase letters (a–d) indicate significant difference between light treatments at the 0.05 level of confidence (one-sided ANOVA, Kruskal–Wallis with Dunn–Bonferroni post-hoc comparison). NL: normal light, HL: high light, FRL: far-red light, LL: low light. MIT: mitochondria, POX: peroxisomes, CHL: chloroplasts, CYT: cytosol, NUC: nuclei.

Col-0	MIT	POX	CHL	CYT	NUC
NL	549 ± 18	169 ± 17	140 ± 4.2	185 ± 5.8	300 ± 15
HL	699 ± 25 + 27% ***	169 ± 23	107 ± 6.9 − 24% ***	157 ± 7.2 − 16% ***	220 ± 10 − 27% ***
FRL	519 ± 18 − 5%	122 ± 10 − 28%	123 ± 9.1 − 12% ***	205 ± 10 + 10%	247 ± 18 − 18% **
***vtc2-1***					
NL	219 ± 8	19 ± 2.8	9 ± 0.5	19 ± 0.7	38 ± 1
HL	191 ±9 − 13%	22 ± 4.1 + 15%	9 ± 0.6	15 ± 0.5 − 21% ***	37 ± 2 − 3%
FRL	236 ± 10 + 8%	53 ± 8.4 + 1.8× ***	16 ± 1.3 + 78% ***	25 ± 1.6 + 32% ***	52 ± 2 + 37% ***
***pad2-1***					
NL	104 ± 7	3 ± 0.9	2 ± 0.2	2 ± 0.2	1.4 ± 0.2
HL	205 ± 10 + 97% ***	9 ± 1.9 + 2× **	3 ± 0.3 + 59% ***	6 ± 0.5 + 2× ***	6 ± 0.5 + 5× ***
FRL	157 ± 09 + 51% ***	7 ± 1.5 + 1.3×	2 ± 0.2 + 35%***	3 ± 0.3 + 94% ***	3 ± 0.3 + 2× ***
**Wheat**					
LL	224 ± 10 − 24% a	31 ± 2 − 48% a	19 ± 2 − 17% a	25 ± 2.2 − 65% a	32 ± 2 − 78% a
NL	295 ± 12 b	60 ± 9 b	23 ± 1.6 a	72 ± 3.6 b	146 ± 05 b
HL	375 ± 24 + 27% c	145 ± 11 + 1.4× c	46 ± 2.4 + 1× b	157 ± 11 + 1× c	271 ± 13 + 86% c
FRL	407 ± 17 + 38% c	117 ± 11 + 95% c	58 ± 3 + 1.5× c	204 ± 10 + 1.8× d	302 ± 8 + 1× d

## Data Availability

The data presented in this study are available in this article (Comparison of Light Condition-Dependent Differences in the Accumulation and Subcellular Localization of Glutathione in *Arabidopsis* and Wheat) and in Supplemental Materials of this article.

## Supplementary Materials

Supplementary materials can be found at https://www.mdpi.com/1422-0067/22/2/607/s1. Figure S1: Expression of genes encoding enzymes of glutathione reduction in leaf extracts of *Arabidopsis* lines grown under various light conditions; Figure S2: Subcellular compartmentation in *Arabidopsis thaliana* Col-0 (a) and wheat (b) mesophyll cells of plants exposed to normal light conditions (white light at 250 µmol m^−2^ s^−1^) without glutathione-specific immunohistochemical labelling; Figure S3: Subcellular distribution of glutathione in *Arabidopsis thaliana vtc2-1* grown under various light conditions; Figure S4: Subcellular distribution of glutathione in *Arabidopsis thaliana pad2-1* grown under various light conditions; Figure S5: Subcellular glutathione distribution in *Arabidopsis* grown under various light conditions; Figure S6: Subcellular glutathione distribution in wheat grown under various light conditions; Table S1: The applied light conditions; Table S2: List of primers used for quantitative PCR reactions; Table S3: Effect of high light and low red/far-red (R/FR) ratio on the amount and redox state of glutathione in *Arabidopsis* and wheat.

## Abbreviations

γ-ECS	γ-glutamylcysteine synthetase
GR	Glutathione reductase
GSH	Reduced glutathione
GSSG	Oxidized glutathione
GSHS	Glutathione synthetase
ROS	Reactive oxygen species
roGFP	Reduction-oxidation sensitive green fluorescent protein
TEM	Transmission electron microscope
