# Supplementary material for: Comparison of Light Condition-Dependent Differences in the Accumulation and Subcellular Localization of Glutathione in Arabidopsis and Wheat
Source: Int J Mol Sci. 2021 Jan 9;22(2):607. doi: 10.3390/ijms22020607 (PMC7827723; doi:10.3390/ijms22020607)
Supplement: Supplementary file 1 [file ijms-22-00607-s001.zip › ijms-1069218-supplementary/Fig. S1.docx]

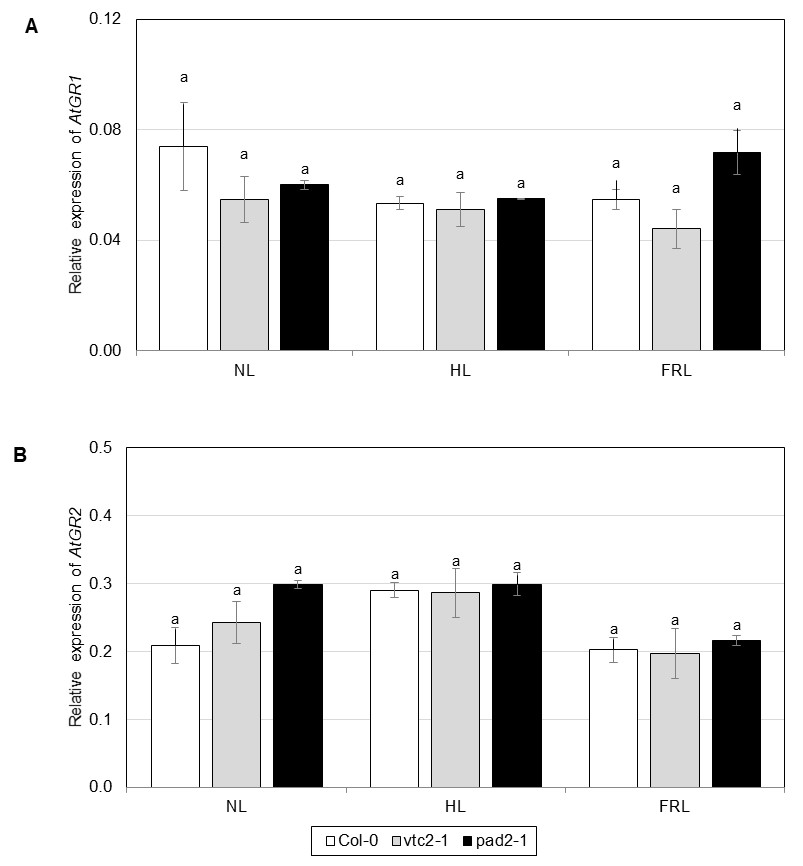


Fig. S1. **Expression of genes encoding enzymes of glutathione reduction in leaf extracts of *Arabidopsis* lines grown under various light conditions.** A: glutathione reductase 1 (*AtGR1*), B: glutathione reductase 2 (*AtGR2*). The experiment was repeated three times with three parallels. Error bars represent standard deviations (*n* = 3). Statistical analysis was performed by one-factor ANOVA. Significant differences at p<0.05 are indicated by different letters above the columns. NL: normal light, HL: high light, FRL: far-red light.
