# Supplementary material for: Comparison of Light Condition-Dependent Differences in the Accumulation and Subcellular Localization of Glutathione in Arabidopsis and Wheat
Source: Int J Mol Sci. 2021 Jan 9;22(2):607. doi: 10.3390/ijms22020607 (PMC7827723; doi:10.3390/ijms22020607)
Supplement: Supplementary file 1 [file ijms-22-00607-s001.zip › ijms-1069218-supplementary/Fig. S2.docx]

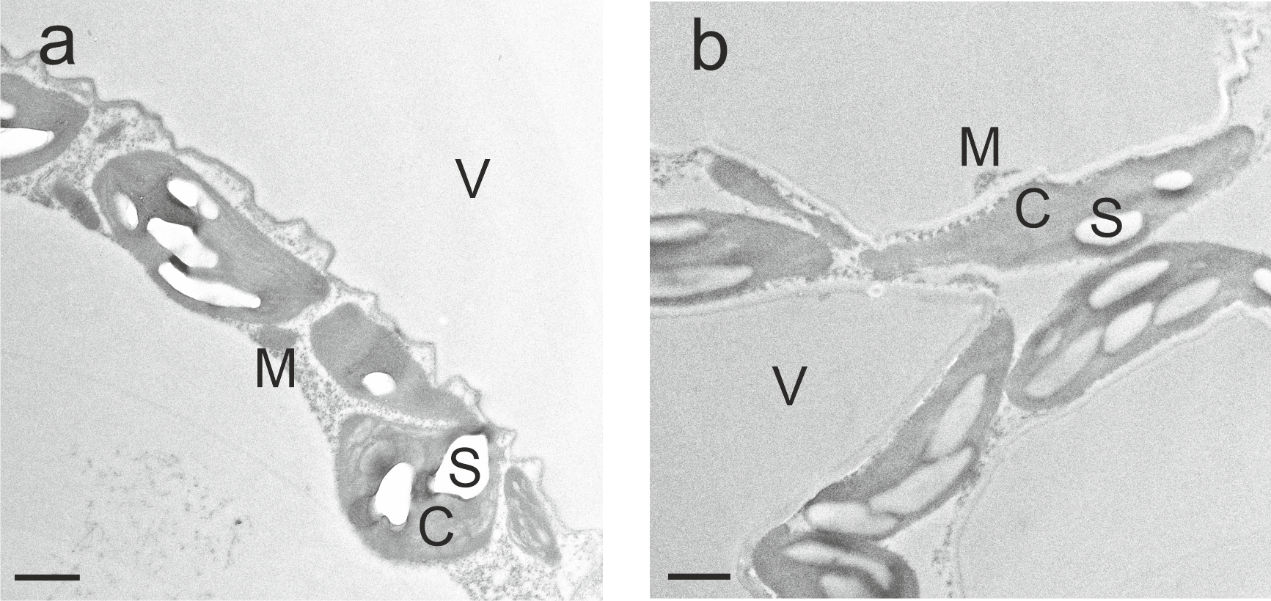


**Figure S2. Subcellular compartmentation in *Arabidopsis thaliana* Col-0 (a) and wheat (b) mesophyll cells of plants exposed to normal light conditions (white light at 250 µmol m-2 s-1) without glutathione-specific immunohistochemical labelling.** Representative TEM images show parts of mesophyll cells. C = chloroplasts with starch (S), M = mitochondria and V = vacuoles. Bars =1µm.
