# Supplementary material for: Comparison of Light Condition-Dependent Differences in the Accumulation and Subcellular Localization of Glutathione in Arabidopsis and Wheat
Source: Int J Mol Sci. 2021 Jan 9;22(2):607. doi: 10.3390/ijms22020607 (PMC7827723; doi:10.3390/ijms22020607)
Supplement: Supplementary file 1 [file ijms-22-00607-s001.zip › ijms-1069218-supplementary/Fig. S4.docx]

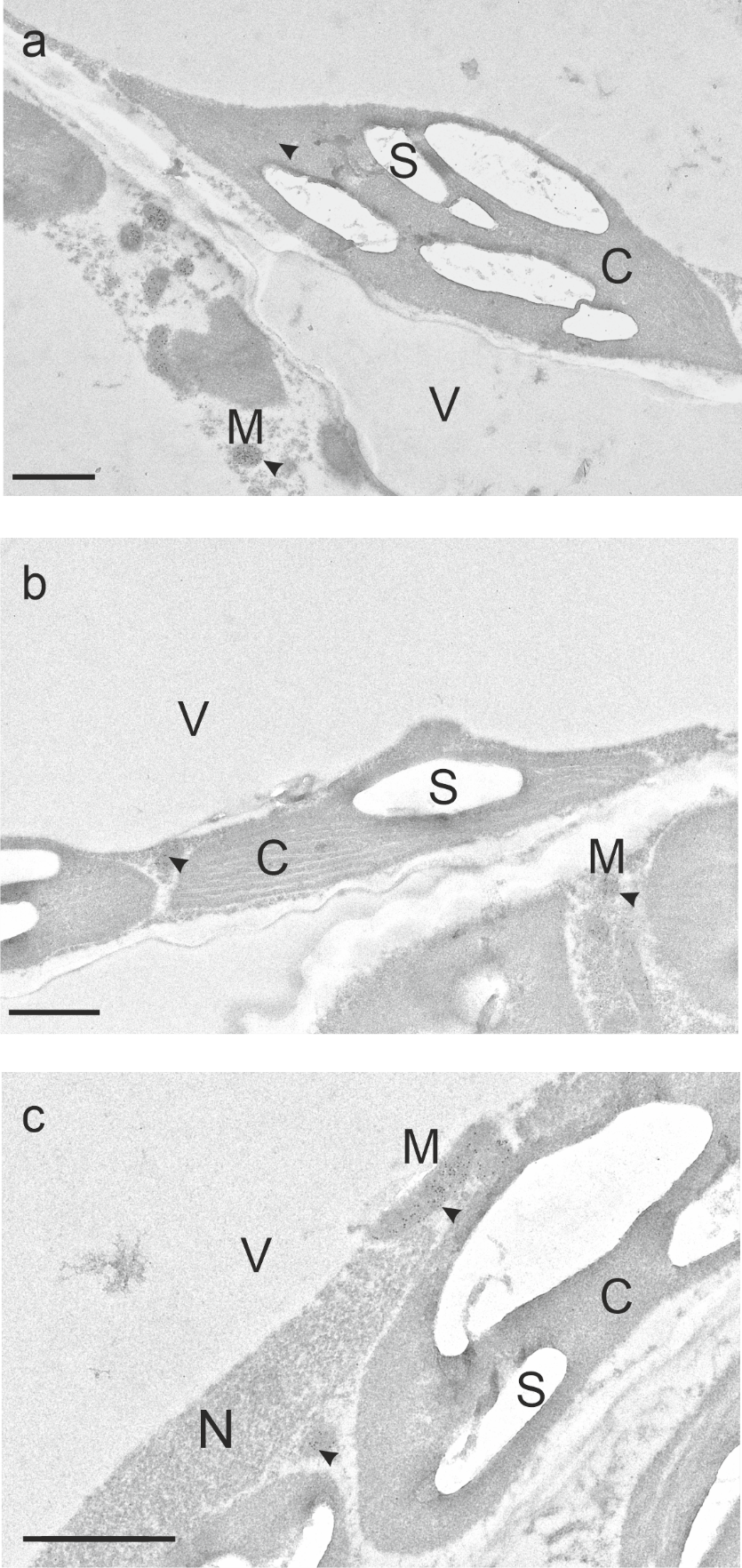


**Figure S4. Subcellular distribution of glutathione in *Arabidopsis thaliana* *pad2-1* grown under various light conditions**. Representative TEM images show compartment-specific glutathione distribution (dark dots, arrowhead) in parts of mesophyll cells from plants exposed to a: normal light (NL), b: high light (HL) and c: far-red light (FRL). C = chloroplasts with starch (S), M = mitochondria, N = nucleus, V = vacuoles. Bars =1µm.
