# Supplementary material for: Comparison of Light Condition-Dependent Differences in the Accumulation and Subcellular Localization of Glutathione in Arabidopsis and Wheat
Source: Int J Mol Sci. 2021 Jan 9;22(2):607. doi: 10.3390/ijms22020607 (PMC7827723; doi:10.3390/ijms22020607)
Supplement: Supplementary file 1 [file ijms-22-00607-s001.zip › ijms-1069218-supplementary/Fig. S5.docx]

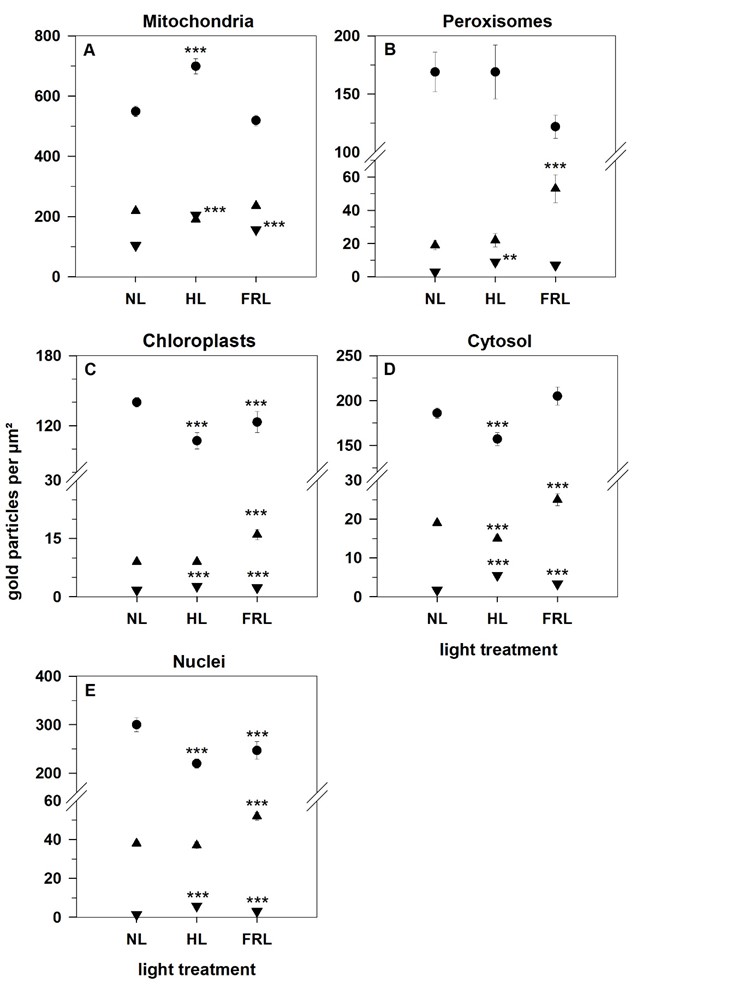


**Figure S5.** **Subcellular glutathione distribution in *Arabidopsis* grown under various light conditions.** Compartment specific means of gold particles bound to glutathione (± SE) per µm² in A: mitochondria, B: peroxisomes, C: chloroplasts, D: cytosol, E: nuclei of *Arabidopsis* (Col-0 black circles, *vtc2-1* black triangles, *pad2-1* black inverted triangles). Values represent the mean of 60 cell compartments, except >11 for peroxisomes. Significant differences between NL and the other treatments (HL, FRL) were compared within the same line (Col-0, *vtc2-1*, *pad2-1*) by the Mann Whitney U-Test. Significance is indicated at 0.01 (**) or 0.001 (***) level of confidence. LL: low light, NL: normal light, HL: high light, FRL: far-red light.
