# Supplementary material for: Comparison of Light Condition-Dependent Differences in the Accumulation and Subcellular Localization of Glutathione in Arabidopsis and Wheat
Source: Int J Mol Sci. 2021 Jan 9;22(2):607. doi: 10.3390/ijms22020607 (PMC7827723; doi:10.3390/ijms22020607)
Supplement: Supplementary file 1 [file ijms-22-00607-s001.zip › ijms-1069218-supplementary/Fig. S6.docx]

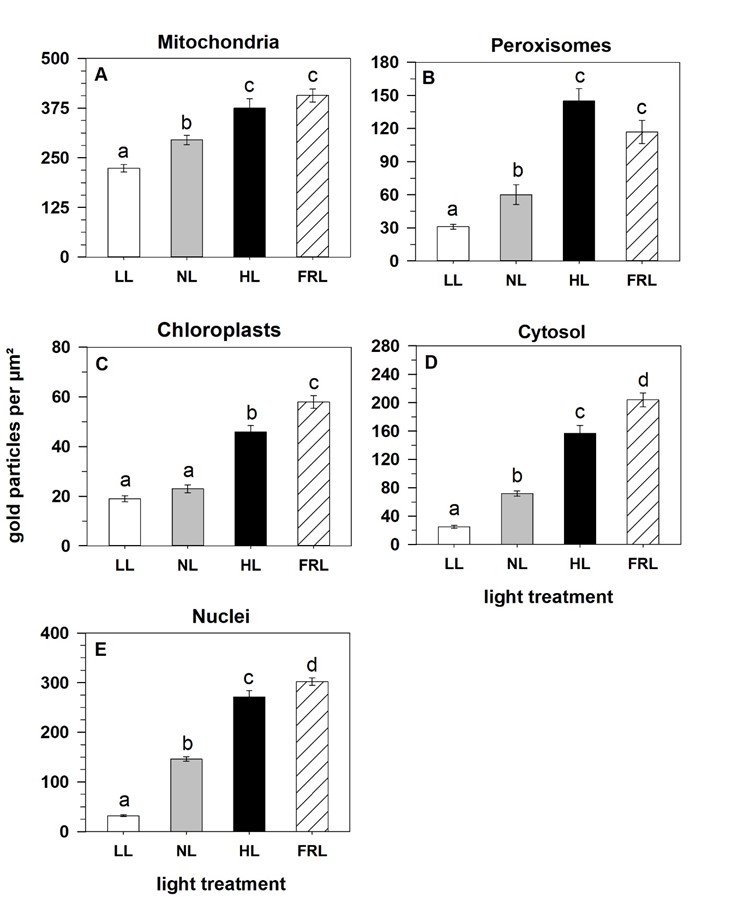


**Figure S6.** **Subcellular glutathione distribution in wheat grown under various light conditions.** Compartment specific means of gold particles bound to glutathione (± SE) per µm² in A: mitochondria, B: peroxisomes, C: chloroplasts, D: cytosol, E: nuclei of *Triticum aestivum* ssp. *aestivum* Chinese. Values represent the mean of 60 cell compartments, except >20 for peroxisomes. Significant differences between NL and the other treatments (LL, HL, FRL) were assessed by one-sided ANOVA (Kruskal-Wallis with Bonferroni-Dunn *post-hoc* comparison). Significant differences between treatments at the 0.05 level of confidence are indicated by different lowercase letters above the columns. LL: low light, NL: normal light, HL: high light, FRL: far-red light.
