# Supplementary material for: Comparison of Light Condition-Dependent Differences in the Accumulation and Subcellular Localization of Glutathione in Arabidopsis and Wheat
Source: Int J Mol Sci. 2021 Jan 9;22(2):607. doi: 10.3390/ijms22020607 (PMC7827723; doi:10.3390/ijms22020607)
Supplement: Supplementary file 1 [file ijms-22-00607-s001.zip › ijms-1069218-supplementary/Table S1.docx]

**Table S1.** The applied light conditions.

|  | Low white | Normal white | High white | Far-red |
| --- | --- | --- | --- | --- |
| PAR (µmol m^-2^ s^-1^) | 50 | 250 | 500 | 250 |
| Blue/Red | 1:2 | 1:2 | 1:2 | 1:5 |
| Red/Far-red | 15:1 | 15:1 | 15:1 | 10:1 |
