# Supplementary material for: Comparison of Light Condition-Dependent Differences in the Accumulation and Subcellular Localization of Glutathione in Arabidopsis and Wheat
Source: Int J Mol Sci. 2021 Jan 9;22(2):607. doi: 10.3390/ijms22020607 (PMC7827723; doi:10.3390/ijms22020607)
Supplement: Supplementary file 1 [file ijms-22-00607-s001.zip › ijms-1069218-supplementary/Table S3.docx]

**Table S3.** Effect of high light and low red/far-red (R/FR) ratio on the amount and redox state of glutathione in *Arabidopsis* and wheat. Fluorescent detection was used for the determination of glutathione content in leaf extracts and immunocytochemistry was applied for the analysis of its subcellular levels. About half of the cellular glutathione content was found in mitochondria and its remaining part was distributed between nuclei, chloroplasts, peroxisomes and cytosol (other organelles). GSH: reduced glutathione, GSSG: oxidized glutathione, TG: total glutathione (GSH+GSSG), mit.: mitochondria, org.: organelle. **↑**: increase, **↓**: decrease, **-**: no change compared to normal light.

|  | ***Arabidopsis*** | | | **Wheat** |
| --- | --- | --- | --- | --- |
|  | wild type | *vtc2-1* | *pad2-1* | Chinese Spring |
| **Fluorescent detection** |  |  |  |  |
| *High light* |  |  |  |  |
| GSH | **↑** | **↑** | **↑** | **↑** |
| GSSG | **↑** | **↑** | **↑** | **↑** |
| GSSG/GSH | **↑** | **↑** | **↑** | **↑** |
| *Low R/FR ratio* |  |  |  |  |
| GSH | - | - | **↑** | **↑** |
| GSSG | - | - | - | **↑** |
| GSSG/GSH | - | - | - | **↑** |
| **Immuno-cytochemistry** |  |  |  |  |
| *High light* |  |  |  |  |
| TG – mit. | **↑** | - | **↑** | **↑** |
| TG – other org. | **↓** | - | **↑** | **↑** |
| *Low R/FR ratio* |  |  |  |  |
| TG – mit. | **-** | **-** | **↑** | **↑** |
| TG – other org. | **↓** | **↑** | **↑** | **↑** |
